# Financial education for HIV‐vulnerable youth, orphans, and vulnerable children: A systematic review of outcome evidence

**DOI:** 10.1002/cl2.1071

**Published:** 2020-02-25

**Authors:** Nanci Lee, Sabrina Beeler Stücklin, Patricia Lopez Rodriguez, Meryem El Alaoui Faris, Ida Mukaka

**Affiliations:** ^1^ Sisters Ink Halifax Nova Scotia Canada; ^2^ Universidad Iberoamericana Mexico City Mexico; ^3^ Stephen Lewis Foundation Toronto Canada

## Abstract

**Problem:**

According to Joint United Nations Programme on HIV/AIDS (UNAIDS) and the World Health Organization, HIV is the leading cause of youth mortality in Africa, and the second cause of death among young people worldwide. Global commitments to reverse the HIV epidemic will only be achieved if strategies prioritize children and youth. Relevant evidence reviews found mixed evidence that HIV prevention may be addressed through economic strengthening activities such as financial education for youth. There was some evidence related to the potential for plural interventions that include both financial and sexual, reproductive health education. However, there is limited quality evidence that focused on HIV vulnerable youth in low‐ and middle‐income countries (LMICs).

**Aims:**

This systematic review assessed the scope and strength of evidence for financial education and plural interventions aimed at reducing HIV vulnerability for youth, orphans and vulnerable children (OVC) in LMICs.

**Methods:**

Standard methodological procedures expected of systematic reviews were used. Six scientific and 24 grey literature sites were searched for relevant studies in English, French, Spanish and Arabic published between 1990 and 2016. Experimental and quasi‐experimental research methods were considered where data was gathered at baseline and at least 6 months after the end of the intervention. Mixed‐methods studies were considered provided they demonstrated validity in terms of randomization, appropriate sampling and controls, and minimization of bias errors and attrition. Evidence was then analysed and mapped to show types of financial and plural interventions by outcome type, direction and strength of evidence through qualitative assessments by the team. In addition, meta‐analysis of odds ratios was conducted to validate the strength of evidence. This analysis illustrated the relative effect or weight of interventions on HIV‐related outcomes based on confidence levels and sample sizes.

**Results:**

Of 5,216 records, 16 moderate to higher quality studies representing 10 interventions were identified, mostly focusing on HIV‐vulnerable girls in Sub‐Saharan Africa. More than half of the interventions were plural and included access to finance and counselling or supports to improve confidence, negotiating ability and social conditions. Most studies used an experimental design. Only 11 of the 16 studies had comparable enough measures to be validated with meta‐analysis of odds ratios.

**Findings:**

The strongest evidence showed plural education interventions with self‐efficacy supports, with and without savings to have positive effects on HIV‐related outcomes. These outcomes included improved knowledge, attitudes and reduced sexual risk‐taking behaviour. Evidence also showed improved self‐efficacy from plural interventions, the changes in confidence, negotiating ability and social conditions that enable people to act on knowledge. Self‐efficacy seems important as both a set of conditions to support reduced vulnerabilities and a way to measure them in terms of outcomes. While positive effects were also observed related to increased savings and improved attitudes toward saving, generally interventions showed mixed effects on financial and economic outcomes.

**Conclusions:**

This systematic review supports emerging evidence that plural interventions are associated with positive health and economic outcomes for vulnerable youth and children in LMIC. Even so, as a body of evidence, it is not clear which components are effective at producing favourable outcomes. Therefore, asset theories linking financial education and asset building with favourable outcomes for vulnerable youth are not as clear as may be commonly assumed. Quality evidence is needed in more settings separating out economic, health and self‐efficacy components to better understand pathways and effects on outcomes. Segmentation in quantitative studies will enhance our understanding of asset, capability and self‐efficacy theories for greater impact. Mixed methods and qualitative studies will be important complements to enhance our understanding of contextual conditions and how to build assets and self‐efficacy in HIV vulnerable youth and OVC.

## 1 PLAIN LANGUAGE SUMMARY

### 1.1 Plural education improves HIV and self‐efficacy outcomes, but the effect on economic outcomes is mixed

#### 1.1.1 Background

According to Joint United Nations Programme on HIV/AIDS (UNAIDS) and the World Health Organization, HIV is the leading cause of youth mortality in Africa, and the second cause of death among young people worldwide. Global commitments to reverse the HIV epidemic will only be achieved if strategies focus on children and youth. There is some positive evidence linking economic interventions and HIV reduction.

#### 1.1.2 What is this systematic review about?

This systematic review summarizes outcome evidence of financial education for young people and plural interventions (financial combined with sexual and reproductive health education) aimed to reduce HIV vulnerability for youth, orphans, and vulnerable children in low and middle‐income countries (LMICs).

#### 1.1.3 What studies are included?

Eligible studies evaluated financial education, with and without savings services, as well as plural interventions with and without social supports (such as mentoring).

Experimental and quasi‐experimental research methods were considered, where data were gathered at baseline and at least 6 months after the end of the intervention.

Sixteen moderate‐ to higher‐quality studies representing 10 interventions were identified, mostly focusing on HIV‐vulnerable girls in Sub‐Saharan Africa. However, a meta‐analysis of odds ratios is restricted to the 11 studies (on six interventions) that had comparable measures.

#### 1.1.4 What are the main findings of this systematic review?

Evidence for plural education interventions with self‐efficacy supports with and without savings—all show a positive effect on outcomes related to HIV‐related knowledge and attitudes, and reduced sexual risk‐taking behavior.

The evidence also shows improved self‐efficacy from plural interventions, the changes in confidence and social conditions that enable people to act on knowledge.

However, while some financial and plural interventions show positive effects on savings and improved attitudes toward saving, generally interventions showed mixed effects on the full range of financial and economic outcomes, such as asset building and engagement in income‐generating activities.

#### 1.1.5 What do the findings of this systematic review mean?

This systematic review supports the growing evidence that plural interventions are associated with positive health and economic outcomes for vulnerable youth and children in LMICs. Even so, as a body of evidence, it is not clear which components are effective at producing favorable outcomes.

Asset theories linking financial education and asset building with favorable outcomes for vulnerable youth are not as clear as may be commonly assumed.

Self‐efficacy is also unclear but seems important as both a set of conditions to support reduced vulnerabilities and a way to measure them in terms of outcomes.

Quality evidence is needed in more settings separating out economic, health, and self‐efficacy components to better understand pathways and effects on outcomes.

Experimental methods are appropriate to segment components for better analysis and understanding. Mixed methods and qualitative methods will be important complements to experimental research, to enhance our understanding of how to build assets and self‐efficacy in HIV‐vulnerable youth, orphans, and other vulnerable children.

#### 1.1.6 How up‐to‐date is this systematic review?

The review authors searched for studies published up to January 2017.

## 2 EXECUTIVE SUMMARY

### 2.1 Background

This systematic review assessed outcome evidence of financial education and plural interventions aimed to reduce HIV vulnerability for youth, orphans, and vulnerable children in LMICs. Relevant evidence reviews found both positive, negative and mixed evidence that HIV prevention may be addressed through economic strengthening activities such as financial education for youth. There was some evidence related to the potential for plural interventions that combine financial education with sexual and reproductive health education but limited quality evidence that focused on outcomes for HIV vulnerable youth in low‐ and middle‐income countries.

### 2.2 Results

To address this gap, PubMed, EconLit, Cochrane Library, Web of Science, Popline, Economia y Negocios, and 24 grey literature sites were searched for relevant studies in English, French, Spanish, and Arabic published between 1990 and 2016 with reference checking and citation searches. The search stopped in January 2017. Eligible studies focused on financial education offered to orphans and vulnerable children and youth in LMICs as defined by the United Nations.^8^ Financial education, with and without finance, as well as plural education (financial combined with sexual and reproductive health education) with and without social supports such as mentoring were included. Experimental and quasi‐experimental research methods were considered where data was gathered at baseline and at least 6 months after the end of the intervention. Mixed‐methods studies were considered provided they demonstrated validity in terms of randomization, appropriate sampling and controls, and minimization of bias errors and attrition.

The team systematically reviewed the scope and strength of impact evidence of financial education and plural interventions for HIV‐vulnerable youth and orphans and vulnerable children (OVC) in LMIC, with a focus on outcome analysis. Standard methodological procedures expected of systematic reviews were used. Of 5,216 records, 16 moderate to higher‐quality studies representing 10 interventions were identified, mostly focusing on HIV‐vulnerable girls in Sub‐Saharan Africa. More than half of the interventions were plural and included access to finance and counseling or social supports. Most used an experimental design. The strongest odds ratio effects were found to be by plural interventions on HIV‐related outcomes. Observed improvements included increased HIV‐related knowledge, attitudes, reduced sexual risk‐taking behavior, and improved self‐efficacy. While positive effects were also observed related to increased savings and improved attitudes toward saving, generally interventions showed mixed effects on financial and economic outcomes. Results are also limited by the 11 studies (on six interventions) that had comparable enough variables and treatment conditions to be validated in the meta‐analysis of odds ratios.

### 2.3 Authors' conclusions

This review supports emerging evidence that plural interventions are associated with positive health and economic outcomes for vulnerable youth and children in LMIC. Even so, as a body of evidence, it is not clear which components are effective at producing favorable outcomes. Asset theories linking financial education and asset building with favorable outcomes for vulnerable youth are not as clear as may be commonly assumed. Self‐efficacy is also unclear but seems important as both a set of conditions to support reduced vulnerabilities and a way to measure them in terms of outcomes. Quality evidence is needed in more settings separating out economic, health, and self‐efficacy components to better understand pathways and effects on outcomes. Experimental methods are appropriate to segment components for better analysis and understanding. Mixed methods and qualitative methods will be important complements to experimental research to enhance our understanding of how to build assets and self‐efficacy in HIV vulnerable youth and OVC.

## 3 BACKGROUND

### 3.1 The issue

Global commitments to reverse the HIV epidemic will only be achieved if strategies focus on children and youth based on United Nations definitions.^1^ HIV is the leading cause of youth mortality in Africa, and the second cause of death among young people worldwide (UNAIDS, 2015; WHO, 2014). Young girls are particularly vulnerable (Fleischman & Peck, 2015), accounting for 20% of new HIV infections globally in 2015, despite accounting for just 11% of the adult population (UNAIDS, 2016, p. 8). As of 2014, 13.3 million children were OVC^2^ by AIDS globally of an estimated 140 million orphaned to all causes (UNICEF, 2016).

### 3.2 The intervention

There is positive evidence linking economic interventions and HIV reduction. Kim, Pronyk, Barnett, and Watts (2008, p. 61) found that economic interventions have more potential to address structural issues such as poverty and gender norms that influence the health context as compared to more conventional health strategies. Orton et al. (2016) found that membership in larger, established microfinance groups showed some improvements in maternal and infant mortality, better sexual health and, in some cases, lower levels of interpersonal violence. Pronyk et al. (2008) found the combination of financial education and microfinance effective at reducing HIV‐risk behavior in young women in rural South Africa. Cui, Lee, Thirumurthy, Muessig, and Tucker (2013) found potential utility in microenterprise development to reduce HIV risk, particularly for sex workers with limited effects for nonsex workers. Another study found household wealth to be a strong predictor of child outcomes for those affected by HIV/AIDS, including wasting, sexual debut, and school attendance (Akwara et al., 2010). Asset theory holds that economic and financial supports reduce economic vulnerability and increase overall resources which may lead to other positive personal and social outcomes (Kennedy, Fonner, O'Reilly, & Sweat, 2014; Witte et al., 2015).

However, other evidence suggests that increased economic strengthening does not necessarily translate to improved health and HIV prevention. Kennedy et al. (2014, p. 13) found positive effects on HIV‐prevention condom use and reducing sexual partners in a minority of studies. There was “inconclusive evidence that microfinance and vocational skills interventions were effective at changing HIV‐related sexual risk behaviors,” noting the inadequate statistical power in some studies. One study even showed an increased risk of harassment. Dworkin and Blankenship (2009) found microfinance to show mixed results in offering young women independence from male partners. However, there was more positive evidence where skills taught in microfinance included assertiveness and challenging gender norms. ICRW (2010) cautioned that economic strategies do not necessarily empower girls and women given social, economic, and legal constraints that are structural in nature. This study also identified that economic activities may increase incentives for girls to leave school which may expose them to different kinds of risks. Students reported positive impacts on self‐confidence but found financial education less applicable when they lived in poverty (Shephard et al., 2017).

There is growing recognition that in HIV‐prevention, economic vulnerability is best addressed alongside other vulnerabilities through multiple interventions, such as health education, counseling, safe spaces, training in negotiating ability, and well‐being (De Guzman, 2001; Dworkin & Blankenship, 2009; Kim et al., 2008; Plourde, Fischer, Cunningham, Brady, & McCarraher, 2016; UNAIDS & The African Union, 2015). The confidence and “self‐efficacy” to act on knowledge is a complex mix of personal and societal norms and conditions (Bandura, 1977; Bandura, 1994). Therefore, the ability to reach economic goals may also be associated with increased self‐efficacy to avoid unprotected sex that some housing and income‐generating arrangements entail (Jennings, Ssewamala, & Nabunya, 2016, p. 279). ICRW (2010) found that social supports and mentoring improved outcomes in their review of eight economic and plural interventions for HIV‐vulnerable girls and young women. Economic stress was linked with low decision‐making power for girls which can lead to early school dropouts, early marriage, and gender‐based violence including trafficking.

### 3.3 Why it is important to do the systematic review

There is emerging but scant evidence related to the particular effects of financial education within these economic interventions for HIV vulnerable youth (Kim et al., 2008) particularly in the Global South with an analysis of outcome pathways. Plural interventions usually have economic, health, and self‐efficacy components. However, too few studies separate these components to understand their particular effects on HIV outcomes (CYFI, 2012; YouthPower, 2017). Further, few impact studies have: adequate sample sizes (Kim et al., 2008); long‐term effects (Kalamar, Bayer, & Hindin, 2016; YouthPower, 2017) reliability and validity related to data contamination, attrition, and statistical power (Kalamar et al., 2016).

This evidence review addresses some of these gaps by focusing on high‐quality evidence for financial education and plural interventions for HIV vulnerable youth and OVC in low‐ and middle‐income countries (LMIC) as World Bank defined.^3^ The review examined intervention components to analyze outcome pathways for reducing HIV vulnerability in youth and children.

## 4 METHODOLOGY

**Figure 1:**
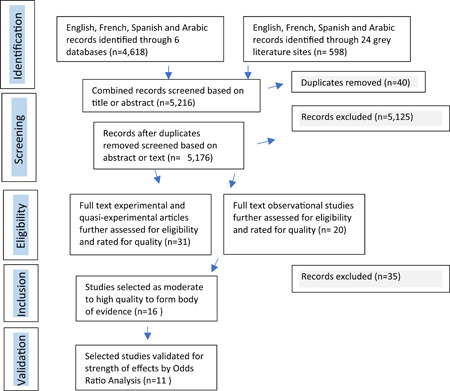
The detailed search process

A diverse team^4^ searched PubMed, EconLit, Cochrane Library, three other economic and health‐related databases^5^ and 24 grey literature sites^6^ for relevant studies in English, French, Spanish, and Arabic published between 1990 and 2016.^7^ See Figure 1 for The Detailed Search Process. Key search words included: financ* education; finance* literacy; economic strengthening; HIV; AIDS; vulnerable; children; youth; OVC (See Appendix 1). Two reviewers screened the titles and abstracts for relevant studies focused on financial education for vulnerable youth or OVC in LMIC with or without financial services.^8^ Plural interventions that combined financial with health, sexual and reproductive education with other social supports were also included. Relevant outcomes included: financial behavior change; financial attitude change; change in use of or access to a financial product or service; change in expenditures related to health; change in treatment, prevention or other behaviors related to HIV; change in the financial situation; change in economic situation; other unanticipated changes in outcomes. Experimental and quasi‐experimental research methods were considered where data was gathered at baseline and at least 6 months after the end of the intervention. Mixed‐methods studies were considered provided they demonstrated validity in terms of randomization, appropriate sampling and controls, and minimization of bias errors and attrition. Single‐group pre–post test design or single‐subject design studies were excluded. Qualitative and mixed‐methods studies were included in the literature review as they provided important insights related to vulnerability in youth and children. However, they were not included in the systematic review of evidence given the focus on outcome analysis.

Two independent reviewers screened records based on the title, and where unclear, abstract and main text. Related and cited papers were sought. The search stopped in January 2017. After exclusions and duplicates were removed, 51 studies were selected for quality assessment.^9^

### 4.1 Criteria for considering studies for the review

Three independent reviewers screened full‐text articles and critically rated the quality of evidence‐based on conceptual rigor (clarity of outcomes, coherence of theory of change); methodological rigor (internal validity, external validity, reliability, cogency) (Department for International Development [DFID], 2014); target population relevance (tailoring to audience and gender awareness); and pedagogical rigor (safe and accessible outreach or delivery mechanisms and learner‐centered approaches). Each study was scored on a scale from zero to 100, with a narrative to back the scoring of these four dimensions (see Appendix 2 for the rating tool). If methodological rigor was a serious problem, overall the financial education program and its impact evaluation would fall below a score of 60 out of 100. High‐quality interventions had to have a clear method for randomization, control and could account for, or at least render explicit, potential risks in selection bias, estimation, spillover or contamination effects. Research projects with positive and negative or no effect results were considered, with different lengths of impact, context, and outreach. In order to minimize publication bias, publications from the blind or indexed journal were not included. Incomplete studies were also not included.

From the 51 studies, 16 studies evaluating 10 interventions rated moderate to higher quality and were selected to form the body of evidence. See Appendix 3 for a summary of results for included studies.

### 4.2 Data and analysis

**Figure 2:**
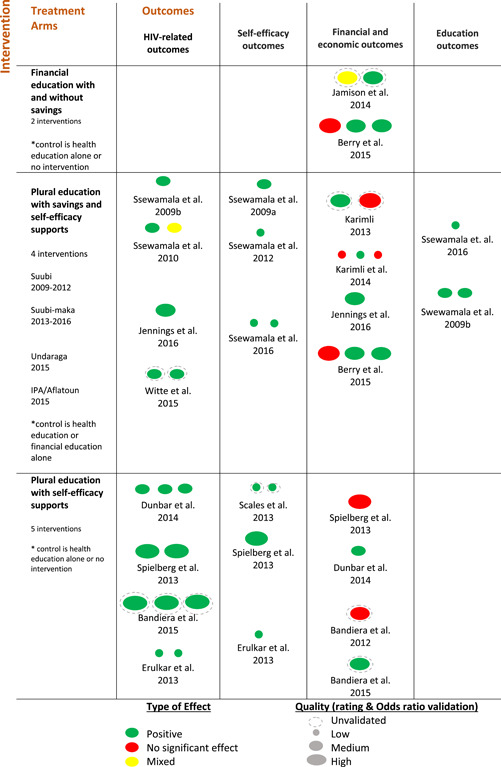
Evidence map

The context mechanisms outcomes (CMO) approach (van der Knaap, Leeuw, Bogaerts, & Nijssen, 2008) was used to map the evidence in terms of multiple pathways to see which programs work.^10^ Context (C) examined the conditions of setting and target population issues including, where available, sex‐disaggregated gender, the average age of participants, setting (school or community), geographic location (urban, rural, peri‐urban). The mechanisms (M) analyzed and clustered the treatment arms to examine individual components of interventions. Outcomes (O) were analyzed by both outcome direction (positive, no effect, mixed) and type (HIV‐related; self‐efficacy; financial and economic; education). See Figure 2 for the evidence map and Appendix 3 for the summary of results and a detailed chart of evidence.

The body of evidence (Appendix 2) was analyzed and mapped based on the rating tool results. In addition, 11 of the 16 studies were also validated using meta‐analysis of odds ratios (Appendix 4). This analysis illustrated the relative effect or weight of interventions on HIV‐related outcomes based on confidence levels and sample sizes. Only 11 studies (on six interventions) had comparable enough variables, standard deviations and treatment conditions to be validated and only data from the baseline and the endline was used. Validated studies included Ssewamala, Han, and Neilands (2009), Ssewamala and Ismayilova (2009), Ssewamala et al. (2010); Ssewamala, Neilands, Waldfogel, and Ismayilova (2012), Ssewamala et al. (2016), Jennings et al. (2016), Karimli, Ssewamala, and Neilands (2014) that correspond to the SUUBI and SUUBI‐Maka project; Dunbar et al. (2014) to the SHAZ! Project; Erulkar, Ferede, Girma, and Ambelu (2013) to Biruh Tesfa Project; Berry, Karlan, and Pradhan (2015) to IPA with Aflatoun and Netherlands Development Organization Project; and Spielberg et al. (2013) to Reach India Project. The outcomes measured are related to HIV attitudes, behavior and knowledge. The independent variable is related to financial education intervention.

The overall measure of effect is represented on the plot as a dashed vertical line (Appendix 4). The graph shows the difference between the intervention and the control group (OR is different than 1). The OR = 1 test reports z‐statistical of 2.47 and *p* value of .014, therefore there is statistical significance to infer that financial education has an impact on HIV incidence, behavior and risk. Results are statistically significant at a 5% significance level.

Studies used cluster randomized controlled trials (CRCT) or randomised controlled trials (RCT), which compare outcome variables between an intervention group and a control group in two periods, a baseline and the intervention endline. In most studies, they combined design terms as randomized controlled, experimental or quasi‐experimental.

### 4.3 Body of evidence

Sixteen studies were included in the body of evidence representing 10 interventions. See Appendix 3 for a summary of results.

In terms of context, eight of these studies alone were related to the SUUBI and SUUBI‐Maka programs in Uganda (representing two interventions). Seven of the interventions took place in Sub‐Saharan Africa and three in Asia. Seven focused on female youth and three on mixed populations. Four of the interventions were in urban settings, four in a rural setting, and two in a mixed setting. Outreach mechanisms or venues included five interventions in community clubs or spaces, four in schools and one in savings groups.

In terms of mechanisms, eight interventions were plural‐focused and two were financial or economic. Berry et al. (2015) was an exception with one intervention arm plural and one intervention arm financial. Since three interventions included more than one arm (Berry et al., 2015; Jamison, Karlan, & Zinman, 2014; Scales et al., 2013), and the latter two studies included more than two arms, the analysis considered arms as well as interventions against the type of outcome. Four interventions were implemented by nongovernmental organizations (NGO) and six interventions were mixed NGO and government.

Eight studies (seven interventions) reported on HIV‐related outcomes, six studies (five interventions) reported on self‐efficacy outcomes, 10 studies (six interventions) on financial and economic outcomes, and two studies (one intervention) reported on education outcomes.

## 5 RESULTS

### 5.1 Summary of main results

This section reports the results of two financial and nine plural education interventions emphasizing the outcome effects. See the evidence map (Figure 2) for the three different types of treatment arms. Broadly, it is possible to examine financial education and plural education arms, with and without savings services and self‐efficacy supports. Each intervention arm shows the corresponding outcome pathway. Each oval represents an individual outcome.

One intervention (Berry et al., 2015) is included in both financial and plural education arms. See Appendix 3 for the summary of results and detailed characteristics of included studies including outcomes effects, statistical test, odds ratios and CIs.

#### 5.1.1 Financial education treatment arms

Two interventions focused on financial education for vulnerable youth (with and without savings) with mixed results on financial and economic outcomes. A study in Uganda combined financial education with savings in one treatment arm and savings‐only in the other (Jamison et al., 2014). The combined treatment group improved financial literacy after 9 months where the savings‐only group did not. These results might indicate that financial education plays a role in the effective use of financial services. However, there was little evidence that savings account access and financial education complement one another.

An intervention in Ghana compared financial education and savings with plural education (combined with personal awareness and child rights education) and savings (Berry et al., 2015). Both led to a positive effect on savings after 9 months mainly due to a change in location (from home to school) rather than an increase. Children were also more likely to work than have leisure with financial education but no negative effect was shown on either child labor or school attendance. These studies show inconclusive evidence for the role of financial education in improving financial literacy and savings.

#### 5.1.2 Plural education with plural supports

Four plural (financial and health education) interventions offered self‐efficacy supports and savings services with six positive effects on HIV outcomes and four positive effects on self‐efficacy. In three of the interventions, plural education led to positive outcomes compared with either health education or financial education alone. The effects on financial and economic outcomes were more mixed with five positive outcome effects and four with no effect.

The earlier intervention in Ghana showed that plural education had the same positive effects on savings behavior (Berry et al., 2015). An intervention with female street‐based sex workers in Mongolia provided financial education and matched savings‐only in the treatment which showed a decrease in the number of paid sexual partners and a decrease in unprotected sexual contacts with paid partners after 6 months (Witte et al., 2015).

The Suubi project targeted HIV‐orphaned and out‐of‐school youth in Uganda. Financial education was offered through their primary schools along with sexual and reproductive health education, matched savings, counseling on life skills, school supplies, and peer mentoring. After 10 months, treatment groups showed positive effects on self‐rated health and sexual risk‐taking behavior as well as improved attitudes toward academic performance and education aspirations (Ssewamala et al., 2009; Ssewamala & Ismayilova, 2009). Later studies showed improved attitudes toward sexual risk for males but not females (Ssewamala et. al., 2010). Positive effects were also found on youth self‐rated mental health (Ssewamala et al., 2009) and depression (Ssewamala et al., 2012).

In terms of economic outcomes, Ssewamala et al. (2010) found no difference in the amount that males and females saved. The value placed on savings and confidence in the ability to save decreased after the matched incentives stopped indicating that these initial attitude improvements may not last. There were no changes in savings attitudes or financial literacy and a few significant effects on risk preferences and spending.

The Suubi‐Maka project followed a similar intervention but extended the treatment groups over 12 and 24 months to include parents to examine family effects on outcomes, which were mixed. While the treatment group reported increased likelihood of the family having saved money (Karimli et al., 2014) and an increase in self‐reported youth savings with some gender differences (Karimli, 2013), later studies showed no significant effect on the reported amounts saved (Jennings et al., 2016; Karimli et al., 2014) and no significant effect on the use of formal financial institutions (Karimli et al., 2014). Savings attitudes were also found to be weakened by the number of children in the family and significantly affected by family relations, family finance, caregivers' gender, adolescent's gender, and educational aspirations (Karimli, 2013). This study seems to indicate that household conditions may affect individual outcomes for youth. HIV preventative attitudes improved as well as attitudes toward family saving, purchases, and vocational training (Jennings et al., 2016).

#### 5.1.3 Plural education with self‐efficacy supports

Five plural interventions offered financial and sexual reproductive health education with self‐efficacy supports and showed positive effects on HIV and self‐efficacy‐related outcomes. All were implemented through groups or clubs often with a peer or trusted adult counseling and personal development supports. Unfortunately, these components were not isolated for analysis of effects on outcomes.

One intervention focused on female youth orphans in Zimbabwe. Financial and health education, vocational training and microgrants were offered to treatment participants where the control arm provided health education and life skills. Positive outcomes included lower risk of transactional sex, higher likelihood of condom use, fewer unintended pregnancies reported, reduced food insecurity, and an improved economic situation compared to the baseline (Dunbar et al., 2014).

One intervention took place in community clubs in rural Uganda with adolescent girls, the majority of whom were in school. They mixed financial education with sexual and reproductive health education and negotiation skills. There was a significant improvement in HIV and pregnancy‐related knowledge, behaviors after 4 years. Sex against their will dropped 26%; early marriage and cohabitation fell by 58%; teen pregnancy fell by 26% (Bandiera et al., 2015). In terms of financial or economic outcomes, results were mixed. An earlier study showed no increased likelihood that treatment participants would engage in income‐generating activities (Bandiera et al., 2012) but a later study showed an increased likelihood mainly driven by increased participation in self‐employment (Bandiera et al., 2015).

An intervention in Ethiopia targeted out‐of‐school girls and youth in slum areas. Supports included HIV awareness training, counseling and testing, self‐esteem, gender and power, basic literacy, and financial education. The control arm had no intervention. This intervention showed positive effects on HIV‐related outcomes after 30 months. Girls in the treatment site were twice as likely to score highly on HIV‐knowledge questions, know where to obtain voluntary counseling and testing, and also more likely to want to be tested. In terms of self‐efficacy, girls in the treatment site were more than twice likely to report social support. The intervention also showed stronger outcome effects on double‐orphaned children where both parents were gone or deceased (Erulkar et al., 2013).

Another intervention aimed to build internal and external assets in vulnerable girls and youth in Bangladesh. Three treatment arms were offered: one health education and school support; one plural adding financial education; and one cooking oil supplement for delayed marriage. The first showed a significant increase in the constructive use of time while the plural intervention showed the largest increase in positive values. External assets overall showed the most significant increases across the treatments. The addition of cooking oil showed a smaller gain in assets (Scales et al., 2013). Unfortunately, the study did not examine health outcomes.

The last plural intervention was implemented in India where adolescent females participated with other women in self‐help groups. While this intervention did not offer savings services, the self‐help groups rotate savings and loans. The treatment groups were provided an array of supports including learning games, health education focused on sanitation, body, and HIV protection as well as financial education and negotiating power. The treatment groups showed significant gains in HIV knowledge including the awareness that condoms prevent HIV and of sexually transmitted infections with some differences depending on the marital status. They also reported self‐efficacy for HIV prevention and confirmed the use of clean needles. There were positive savings outcomes as well as where the girls reported significantly higher motivation to save money and to be more likely to have a plan to save after 1 year (Spielberg et al., 2013).

## 6 DISCUSSION

A number of lessons can be learned from the selected studies on the mix of financial and plural education and supports that led to positive health and economic outcomes for vulnerable youth and children. Both financial and plural education intervention arms showed at least one positive outcome.

### 6.1 Summary of findings

There is some evidence in support of asset theory; that is, linking financial education to asset building for vulnerable youth and children in LMIC but outcome pathways are inconclusive.

The two financial education arms showed mixed results, with positive effects on spending, risk preferences, and savings behavior (Berry et al., 2015) and mixed results on financial literacy and the role that savings play (Jamison et al., 2014).

Three intervention arms that separated financial education from health education in treatment showed the potential value‐added of financial education for HIV and self‐efficacy related outcomes (Dunbar et al., 2014; Scales et al., 2013; Witte et al., 2015). However, treatments differed across these interventions as will be outlined in the next section.

The value‐added of finance is also unclear. The only study to isolate finance from other components (Jamison et al., 2014) found that savings accounts on their own did not increase financial literacy. There was little evidence that account access and financial education were strong complements. In fact, there was some evidence that they were substitutes. Financial education on its own led to an increase in financial literacy. There was no evidence analyzing the effect of finance within plural interventions. Positive effects on outcomes were found both in plural interventions offering and not offering finance.

Four plural education arms, with savings and self‐efficacy supports, showed more positive outcomes than either financial or health education approaches on their own. Observed improvements included increased HIV‐related knowledge, attitudes, reduced sexual risk‐taking behavior, and improved self‐efficacy. While positive effects were also observed related to increased savings and improved attitudes toward saving, the relationship between the economic and health components was less clear. Self‐efficacy supports such as counseling, mentoring, negotiation, and self‐esteem training were also present with positive outcomes but not isolated in the analysis. Studies did not increase understanding of the relationship between positive HIV and self‐efficacy outcomes.

In terms of context and target population, there is some evidence that household‐level conditions and outcomes can affect child and youth outcomes positively (Karimli, 2013). Individual studies found differences in outcomes by marital status (Spielberg et al., 2013), gendered savings attitudes (Karimli, 2013), sexual risk‐taking (Ssewamala et al., 2010), and type of orphan (Erulkar et al., 2013).

### 6.2 Overall completeness and applicability of evidence

This systematic review highlights the evidence and lack of evidence related to the outcome effects of financial and plural education programs on HIV vulnerable children and youth.

The strength of these findings is limited by the 16 studies included in the body of evidence representing 10 interventions. Half of the studies that comprise the body of evidence are profiling two programs, the SUUBI and SUUBI‐Maka programs in Uganda. Even though the focus and conditions of the second program and studies are different, they are a continuation of the earlier program. Even though positive effects on outcomes were found for both financial and plural intervention arms, the heterogeneity in intervention arms, intended outcomes, settings, and targeted populations limit the strength of the body of evidence overall.

Three intervention arms that separated financial education from health education in treatment showed the potential value‐added of financial education for HIV and self‐efficacy related outcomes (Dunbar et al., 2014; Scales et al., 2013; Witte et al., 2015). However, treatments differed across these interventions.

Disaggregated outcome evidence is also thin. Conditional factors such as the setting of the education, whether community or school, if a trusted adult offered counseling were not analyzed.

Because of the focus on disaggregating component interventions and outcomes, the review focused on studies using experimental and quasi‐experimental methods. The review did not include relevant qualitative and mixed‐methods studies as they did not examine outcome pathways. Since this body of evidence is limited to experimental and quasi‐experimental research, it is important to complement this analysis with mixed methods and qualitative studies. These provide contextual grounding for the quantitative studies and elucidate the nuances in outcome pathways inherent in understanding how economic and other vulnerabilities interact.

### 6.3 Quality of the evidence

The review included a comparison of financial education programs with plural programs that included education on sexual and reproductive health. The team drew only on published studies and largely self‐reported data from limited settings. Furthermore, length, number of waves and varied outcome variables and treatment formats of the studies made comparisons and clustering of pathways difficult. Quality of the evidence was assessed, first through a rating of conceptual and methodological rigor as well as relevance to the target population and pedagogical rigor. Internal validity was an issue common across the studies so this aspect was analyzed further. Finally, the meta‐analysis of odds ratios was done to validate the strength of the evidence. Only eleven studies (representing six interventions) had comparable enough treatment variables and setting conditions.

As the evidence map (Figure 2) shows, the quality of the studies included is mixed and some were not validated with the meta‐analysis of odds ratios. The quality of the studies related to financial education arms was high though the Ugandan study was invalidated by the meta‐analysis because the study used Ordinary Least Squares method and did not use comparable variables like odds ratios.

The quality of the evidence for plural education with savings and self‐efficacy supports was mixed. All but two studies, related to the SUUBI and SUUBI‐Maka program in Uganda and three rated low due to internal validity issues and small sample sizes. Only four studies (Ssewamala et al., 2010; Karimli, 2013; Jennings et al., 2016; Witte et al., 2015) report Cronbach's alpha, a measure of internal consistency. However, Karimli (2013) and Witte et al. (2015) were not validated with the meta‐analysis as they did not report comparable variables or setting conditions on the secondary data.

The quality of the evidence for plural education with self‐efficacy supports is also mixed. Two studies were not validated with meta‐analysis due to lack of comparability (Bandiera et al, 2015; Scales et al., 2013). It is unfortunate to not have further validation of these studies as they rated high and low quality, respectively. In addition, the evidence in three of the studies was weakened by small sample sizes and CIs. Spielberg et al. (2013) satisfied the expected criteria for internal validity including estimating intent to treat (ITT). However, in this intervention reporting on community analysis of women's self‐help groups women engaged in sex work willing to participate were self‐selected. In both Spielberg et al. (2013) and Dunbar et al. (2014), there is a risk of self‐selection bias.

The meta‐analysis of odds ratios estimates the strength of evidence and heterogeneity of studies through examination of width and length of CI, the statistical significance of the difference between the intervention and control groups and the heterogeneity or variability between studies. Meta‐analysis synthesize the studies data into a single quantitative estimate, see Appendix 4 for the meta‐analysis of odds ratios graph and chart. The odds ratios output table gives the numerical results and weights of the analysis, the OR column shows the odds ratio and 95% confidence intervals (CI) of each study, which displayed on the meta‐analysis of odds ratios graph.

The calculated difference between the intervention and control groups can be considered as statistically significant, the statistical significance of the overall result is I^2^ = 64.8%, *p* = .002, the *p* value is *p* < .05, therefore the overall result is statistically significant.

The horizontal lines (whiskers) through the boxes depict the length of the CI. The longer the lines, the wider the CI, the less precise the study results, as the case of Ssewamala et al. (2016), contrary to Spielberg et al. (2013) and Berry et al. (2015), which present narrow CI. The analysis of weights indicates the influence of each study on the overall results. In this analysis, Spielberg et al. (2013) and Berry et al. (2015) are the most weighted studies. The higher the percentage weight, the bigger the box, the more influence the study has on the overall results. Since the influence of a study on the overall results is determined by the study's sample size and the precision of the study results provided as a CI, the bigger the sample size and the narrower the CI, the greater the weight of the study is, as Spielberg et al. (2013) and Berry et al. (2015) studies.

Heterogeneity measures the variability between studies, this describes the percentage of variation across studies, an indication of how comparable studies in the analysis are if the 95% CI overlap 1 the studies are regarded as homogeneous, as the cases of Ssewamala et al. (2009), Ssewamala and Ismayilova (2009), Ssewamala et al. (2010), Ssewamala et al. (2012), Ssewamala et al. (2014), Karimli et al. (2014), and Jennings et al. (2016), which estimate results for the same project with different samples size, for these studies the 95% CI overlap 1. Since I^2^ = 64.8% in this analysis (I^2^ < 75%), we used a fixed‐effect model.

The results are with the caveat that format data is not the same in all the studies, (percentage, levels, ratios, coefficients (beta) for a regression analysis). Studies do not use the same HIV outcome variables. Some studies reported more than one dependent variable, or used three waves. For comparability, only the first and the last wave were used. Data used to generate the forest plot odd ratio graph come from the reported in the studies but not from the primary source of the authors (not raw data).

### 6.4 Agreements and disagreements with other studies

Practitioner studies and evidence reviews using qualitative and mixed‐methods helped to inform the analysis in this review by providing nuance to the analysis around vulnerabilities, capabilities, and self‐efficacy (CYFI, 2012; O'Prey & Shephard, 2014; Shephard et al., 2017; YouthPower, 2017). In a longer report, these conceptual frames were explored in more detail but were not within the scope of this article.

The evidence reviews and studies of closest relevance to financial education and youth were conducted by Aflatoun, a Dutch organization, which has developed one of the most widely used financial education curricula combining financial and social education, particularly for children and youth. Aflatoun carried out two systematic reviews on the topic of financial education for children and youth in 2014 and an independent study in 2017 as well as a global desk review of financial education's contribution to girl's economic empowerment. The 2014 qualitative review showed some positive effects. However, 11 of the 21 studies were located in the United States and the review was not specifically focused on vulnerable populations (O'Prey & Shephard, 2014).

The 2017 Aflatoun review (Shephard et al., 2017) aimed to understand programmatic evidence in terms of the effectiveness and sustainability of social and financial education for youth. The study found strong evidence in both the quantitative meta‐analysis and the qualitative data synthesis related to young people's future orientation, resource management behavior (especially savings), entrepreneurship attitudes, and behaviors and positive trends related to rights orientation and gender attitudes, particularly in the qualitative analysis. Qualitative data synthesis also showed positive impacts on self‐confidence (especially for young women) and financial knowledge. Qualitative findings revealed that students felt that the financial component of the curriculum is less applicable when they live in poverty. The sustainability analysis showed over 70% of partner organizations have continued based on the first 4–5 years of the program. Program fidelity was found to be an important driver of sustainability (Shephard et al, 2017, p. 2). Unfortunately, this review did not focus on health outcomes.

Aflatoun's desk review, however, provided valuable contextual and qualitative information about financial, social and health outcomes. The review supplemented a traditional systematic search with purposive sampling and diversity of primary studies to contribute to the overall richness of evidence. The review found that financial outcomes are largely situated within social and health outcomes, or social and health outcomes become an intermediate step to successfully arrive at the financial outcomes. In fact, none had a stand‐alone financial component (Singh & Schneiders, 2017, p. 20). The scope of the review, however, was not to assess the impact of the outcomes or the effect size of the studies. Rather, the review analyzed trends across the theories of change and program models of high‐quality studies based on relevance and rigor. For example, they found that in 10 of the 12 studies, financial education was a secondary component and 11 used a “pluralistic model” combining financial education with health or social education as compared to the one that used a complementary model of combining financial education with other complementary economic components such as financial services, vocational training, and entrepreneurship training.

Aflatoun's body of growing research demonstrates the importance of qualitative studies to provide contextual grounding for the quantitative studies and to elucidate the contextual and programmatic nuances inherent in understanding how vulnerabilities interact. The desk review highlighted the importance of understanding the layers of conditions that are necessary to support girls' economic empowerment from the recognition of girls' value and acceptability of wage labor at the household level to structural inequalities such as restricted mobility and paucity of time. Qualitative and mixed‐method evaluations may be important complements to experimental and quasi‐experimental evaluations that tend to be highly focused on specific variables and causal pathways.

Reviews focused on the links between financial education or economic empowerment and positive youth outcomes found generally positive results. Child and Youth Finance International reviewed interventions aimed at improving economic citizenship and financial capability for children and youth. The studies reviewed suggest that initial evidence shows positive economic, social, and health outcomes for youth (CYFI, 2012). Also, a systematic review of positive youth development (PYD) programs in LMICs found a number of high‐quality studies of health‐focused PYD programs show improved knowledge, attitudes, and behaviors related to sexual and reproductive health. Though limited, some PYD programs have led to positive shifts in gender norms and led to the economic and social empowerment of youth (Youthpower, 2017). The CYFI working model suggests that offering financial education and financial inclusion (services) together may yield more positive results than pursuing one strategy at a time. It is important to mention that almost no research studies are clear‐cut tests of this idea for adults or children and youth especially in developing countries (Youthpower, 2017, p. 12). Kim et al. (2008) note that few studies have focused on vulnerable youth and those that have were not successful with these target groups.

## 7 AUTHORS' CONCLUSIONS

This review supports the emerging evidence that plural approaches (financial education combined with sexual and reproductive health education and self‐efficacy supports) may reduce HIV vulnerabilities in vulnerable children and youth in LMIC. There is a small body of evidence on which to build and refine practice, research, and policies. Nevertheless, the evidence is scant, difficult to compare and quality mixed. While positive effects have been shown across a number of studies, it is often not clear which components are effective at producing favorable outcomes.

One of the challenges of building the body of evidence is, ironically, the very plural approach that seems to be developing the base of evidence. The bundling of financial education with such diverse services such as other forms of education and awareness‐building, economic, and health‐related support services makes impact monitoring challenging. Some are highly focused such as sexual and reproductive health education. Others focus on rights awareness and negotiating skills. Also, outreach mechanisms are very much part of the intervention given outreach through a group, or a home or a community center with either peer or trusted adult mentoring may help to provide the social supports and protective assets that form part of self‐efficacy for vulnerable youth and children. While the bundling of these intervention components may be more effective for certain outcomes, it is difficult to parse out which aspects of the population, the contextual and household conditions, program design and outreach lead to favorable outcomes. While plural approaches seem to be important to address the multiple and reinforcing aspects of HIV vulnerabilities, monitoring outcome pathways becomes challenging with so many components.

Financial education seems important combined with sexual and reproductive health education for vulnerable youth and children but there is little evidence to understand the relationship between the two forms of education and favorable outcomes. Financial education arms even had mixed effects on financial and economic outcomes for the two studies examined and the value‐added of finance is unclear. Overall, whether financial or plural education, there were mixed effects on financial and economic outcomes.

Asset theory pathways linking economic strengthening to reduced vulnerabilities are not as clear as may be commonly assumed for vulnerable children and youth. There is a need to better understand outcome pathways and the mechanisms through which both economic empowerment and bodily integrity or autonomy for HIV‐vulnerable youth and children are achieved.

Self‐efficacy, both as a condition (to be supported) and as a set of outcomes, seems critical to these pathways but details are unclear. The four interventions that aimed for self‐efficacy outcomes had moderate to low quality studies. Household conditions and, arguably, self‐efficacy supports such as counseling, peer mentoring, negotiation, and self‐esteem training were present in studies with positive outcomes but not isolated in the analysis. Likewise, the outreach mechanism or setting, whether in community, schools or savings groups was not isolated for analysis. Studies did not increase understanding of the relationship between positive self‐efficacy and positive HIV outcomes.

Single studies show that gender, age, circumstances of orphanage affect outcomes. However, literature and emerging evidence support that self‐efficacy is more complex than personal well‐being. Negotiating power and the ability to build economic security and retain assets, protect bodily integrity are linked to personal self‐esteem and structural elements such as vulnerabilities that limit choice, social supports, living and livelihood conditions, gendered social norms.

### 7.1 Implications for practice and policy

Plural approaches (financial education combined with sexual and reproductive health education and self‐efficacy supports) have shown the potential to reduce HIV vulnerabilities in vulnerable children and youth in LMIC. In order to better understand pathways to build economic security, assets, bodily integrity, and self‐efficacy, it is critical to understand what sets of conditions, programs, and policy levers lead to reduced vulnerabilities for children and youth in a structural context of limited options.

At a policy level, it is important to ask what types of evaluations and research are needed to fill the gaps in evidence around promising practices.

Experimental research and evaluation have an important role to play. Experimental and quasi‐experimental studies, as found in this review, help the broader economic and health sectors to isolate and analyze intervention components. For these health and economic practitioners and researchers, much greater attention needs to be paid to understanding what particular components lead to favorable outcomes. In particular, it would be helpful to better understand the target population (by age, gender, type of orphan), effectiveness of different program components (whether school, community or savings group setting) what aspects of family or household conditions contribute, what kind of counseling or mentoring supports and by peers and/or trusted adults.

However, as the earlier literature review and additional studies illustrated, mixed methods and qualitative analyses that permit a nuanced understanding of context may be important complements to the highly specific and localized experimental research. Mixed methods and qualitative studies have helped to better understand self‐efficacy, structural issues, and the inter‐relatedness of economic vulnerabilities with health and other vulnerabilities. While there is agreement about the complexity of vulnerability, building assets, and self‐efficacy, much more needs to be understood about how these vulnerabilities are reduced in the face of personal and structural barriers.

Single settings, single approaches to programming or single methods of research will not support the nuanced understanding that is necessary to reduce vulnerabilities in HIV‐vulnerable youth and OVC. Donors and policymakers would do well to continue to fund and support cross‐disciplinary, cross‐sectoral, and multi‐methods research in these areas. Such complexity demands our most creative and collaborative synergies to fill the global gap in this emerging but important body of evidence.

## ROLES AND RESPONSIBILITIES

**Content:** Nanci Lee, Sabrina Beeler Stücklin, Patricia Lopez Rodriguez

**Systematic review methods:** Nanci Lee, Sabrina Beeler Stücklin, Meryem El Alaoui Faris

**Statistical analysis:** Patricia Lopez Rodriquez

**Information retrieval:** Nanci Lee, Sabrina Beeler Stücklin, Patricia Lopez Rodriguez, Meryem El Alaoui Faris, Ida Mukaka

## SOURCES OF SUPPORT

This work was supported by FHI 360 with funding from the United States Agency for International Development (USAID).

## DECLARATIONS OF INTEREST

The authors have no conflicts of interest to declare.

## PLANS FOR UPDATING THE REVIEW

The review will be updated when there are a sufficient number of new studies available, provided the authors are able to attract the necessary resources for doing so.

## Footnotes

1 Children and youth are defined as those between 0 and 24 years by UN and UNICEF definitions (http://www.un.org/esa/socdev/documents/youth/fact‐sheets/youth‐definition.pdf, https://www.unicef.org/crc/files/Guiding_Principles.pdf, retrieved on November 15, 2017).

2 OVC, as defined by PEPFAR (U.S. President's Emergency Plan for AIDS Relief), are children aged 0–17 years who have lost a parent to HIV/AIDS, who are otherwise directly affected by the disease, or who live in areas of high HIV prevalence and may be vulnerable to the disease or its socioeconomic effect (PEPFAR, 2012)

3 As of July 1, 2016, low‐income economies are defined as those with a GNI per capita, calculated using the World Bank Atlas method, of $1,025 or less in 2015; lower‐middle‐income economies are those with a GNI per capita between $1,026 and $4,035.

4 Two academics, one practitioner in the health sector, one practitioner in the finance sector, one HIV organizer.

5 Web of Science, Cochrane Library, EconLit, Popline, PubMed, Economia y Negocios.

6 Aflatoun, Global Partnership for Financial Inclusion and Alliance for Financial Inclusion (AFI), 3ie, MEDA, MasterCard Foundation, United Nations Capital Development Foundation‐YouthStart, World Health Organization, Microfinance Opportunities, Women's World Banking, International Labor Organization, Freedom from Hunger, Save the children—Youthsave, FHI 360, Population Council, Microsave, Child and Youth Finance International, International Gateway for Financial Education (OECD), Google Scholar, UNDP, World Bank, Sultan Qabus University of Oman, Jordanian Public University Library, King Faysal University‐Saudi.

7 The team searched back to 1990 due to limited studies that met criteria.

8 Two programs included adults. The Indian program focused on self‐help groups that included adolescent girls with their mothers but did not target them. Another program focused on sex workers at least 18 years old. Sixteen of the studies focused on girls or women vulnerable to HIV, gender‐based violence including early marriage, trafficking.

9 Reasons for exclusion were: no use of controls, high‐income country, evidence of selection bias, outcomes‐focused only on teachers rather than students; qualitative studies with no controls or other rigor issues, such as obvious or reported issues with selection bias, contamination or spillover risks; evaluation too soon after end of program, vocational entrepreneurship training without clear financial education; protocol or baseline assessments; too little information on which to analyze study.

10 CMO is a realist inquiry that attempts to understand why a particular program or policy works sometimes and not others. In contrast to a results chain, this approach explores multiple pathways (van der Knaap et al., 2008). Van der Knaap et al. (2008) discuss a systematic review process which combines Campbell standards of systematic review with the CMO principle as a basis for aggregation.

## APPENDIX 1 SEARCH TERMS

### Web of Science

“financial education”; financ* literacy; (“financ* education” OR “financ* literacy” OR “financ* training” OR “financ* capabilities” OR “product awareness training” OR (“consumer protection” AND micro*) OR “microenterprise develop*” OR “entrepren* training “ OR “entrepren* development” OR “economic strengthening”) AND ((influenc* OR effect OR develop* OR empower* OR improv* OR test* OR *experiment* OR evidenc* OR grow* OR matter* OR outcome OR impact OR result* OR health OR financ* behavior OR HIV OR AIDS OR economic* strengthening OR behavior OR power) OR (HIV OR AIDS OR (HIV AND (diagnosis OR “risk population” OR youth OR gender OR prevention)) OR OVC OR (“sex workers”) OR “commercial sex” OR “transactional sex”)

### EconLit

"financ* education" OR "financ* training" OR "financ* literacy" OR "financ* capabilit*" OR "entrepr* develop*" OR "economic strengthening" AND influence or impact or effect or affect or develop or enhance or promote OR grow* OR empower* OR improv* OR test* OR experiment* OR evidence OR matter OR outcome OR result* OR health OR "financ* behavior" OR power OR empower*

### Popline

"financial literacy" OR "financial training" OR "financial education" OR "financial capability" OR "financial capabilities" OR "financial behavior" OR "financial behavior" OR "economic strengthening

### PubMed

"financial education"[All Fields] OR "financial literacy"[All Fields] OR (inancial[All Fields] AND ("education"[Subheading] OR "education"[All Fields] OR "training"[All Fields] OR "education"[MeSH Terms] OR "training"[All Fields])) OR “micro finance” OR microfinance OR “micro credit” OR microcredit OR microcredit

### Economia y Negocios

Educación Financiera AND evaluación de impacto OR PSP OR pruebas controladas por aleatoriedad OR pruebas controladas por aleatoriedad por grupos OR regresión discontinua OR Educación Financiera Educación AND pobre Educación Financiera AND remota OR Financiera OR Educación Financiera AND niños AND en la parte más baja de la desigualdad OR Educación Financiera AND población vulnerable OR Educación Financiera AND jóvenes R en los últimos deciles de la distribución Entrenamiento financiero AND America Latina AND Sida, VIH población infectada AND América Latina

### Grey Literature (English)

“financial education” OR “financial literacy” OR “financial capability” OR “economic strengthening”

OR “microfinance” OR “microenterprise” AND “youth” OR “children” OR “HIV youth” OR “AIDS”

### Grey Literature (Spanish)

Financial Education AND Latin America OR Argentina OR Bolivia OR

Brazil OR Chile OR Colombia OR Costa Rica OR Cuba OR Ecuador OR El Salvador OR

Guatemala OR Haití OR Honduras OR Jamaica OR México OR Nicaragua OR

Panamá OR Paraguay OR Perú OR República Dominicana OR Trinidad y Tobago OR

Uruguay OR Venezuela Educación Financiera AND America Latina

Educación Financiera AND evaluación de impacto OR PSP OR pruebas controladas por aleatoriedad OR pruebas controladas por aleatoriedad por grupos OR regresión discontinua OR Educación Financiera Educación AND pobre Educación Financiera AND remota OR Financiera OR Educación Financiera AND niños AND en la parte más baja de la desigualdad OR Educación Financiera AND población vulnerable OR Educación Financiera AND jóvenes R en los últimos deciles de la distribución Entrenamiento financiero AND America Latina AND Sida, VIH población infectada AND América Latina

## APPENDIX 2 QUALITY RATING TOOL FOR ELIGIBLE STUDIES

Each study was scored on a scale from zero to 100, with a narrative assessment to back the scoring. The following is a description of the elements of quality that were assessed: 11 Each parameter of quality was associated with three key questions which were rated either weak (1), moderate (2), strong (3) or not enough information (NA). The total possible score was 36 or less if elements were missing such as curriculum to assess pedagogical rigor. The score was then turned into a % out of 100.

1. Conceptual rigor examined the clarity and coherence of the theory of change, clear measurement of changes in outcomes, and evidence of behavioral outcomes including how they were measured.
2. Methodological rigor used Department for International Development, UK (DFID)'s Quality of Evidence Framework (2014) to assess key principles of high‐quality research.
  - Internal validity. Appropriateness of methodology to research questions. Minimization of risk and bias errors including incomplete data, sampling, and controls. These include selection bias, randomization errors, problems of endogeneity.
  - External validity. Generalizability. Can results from one setting apply to another? This should be asked of all research projects for which there are multiple countries. Also considered are coherence and comparability questions.
  - Reliability. Appropriateness of methodology to what is being assessed. For example, RCTs are best to address the causal, observable phenomenon.
    Also, methods and instruments that will ensure consistent results across tools, contexts, and so on.
  - Cogency. High‐quality studies will provide a clear logical thread that runs through the entire paper. High‐quality studies will guide the reader and avoid making claims in their conclusions that are not clearly backed up by the data and findings. They also identify the limitations of the work and alternative interpretations of analysis.

The review team included questions related to how well the methodology matched the research questions and how robust the impact methodology was in terms of rendering limitations explicit. Because methodological rigor is so foundational to a high‐quality study, the review team gave this area an extra weighting by a factor of five. This weighting was tested across the assessments. As intended, a high rating in areas of pedagogical rigor and relevance did not falsely distort the rigor of the overall program. If methodological rigor was a serious problem, overall the financial education program and its impact evaluation would fall below a score of 60 out of 100.

3. Relevance examined demonstration of tailoring to the audience, analysis of the target group in the context, and clear gender awareness built into design.
4. Pedagogical rigor examined the extent to which curriculum was learner‐centered and tailored to vulnerable children and youth, including safe and accessible outreach or delivery mechanisms.

The following rating tool was used to assess the quality of the impact evaluations.

### Quality rating tool for financial education interventions

Financial Education Intervention (Country):

Corresponding Study (Authors, year):

Reviewer:

## APPENDIX 3 CHARACTE RISTICS OF INCLUDED STUDIES

**Financial education intervention impact studies and outcome effects on vulnerable youth and OVC.**

**Table:**

Intervention and impact evaluations	Funding sources	Context		Intervention			Outcomes	
		Target population	Geographical	Treatment arms and controls	Study design	Length of impact follow‐up following intervention	Outcome effects	Odds ratios and confidence intervals (CI)/
							+ Positive	F, Z outputs (reporting data of the results)
							− Negative	
							= No effect	
			Context					
			Outreach					
IPA, Finca Uganda (Jamison et al., 2014)	Financial Education Fund from DFID	2,680 female youth	Mbarara district Uganda School, 15‐hour course was delivered over 10 weekly meetings	T1: Financial education T2: Access to group accounts (through FINCA) T3: Both C: No intervention	RCT	9 months	+ Financial education increased financial literacy − Account‐only treatment does not increase financial literacy − Little evidence that account access and financial education are strong complements. Some evidence that they are substitutes.	Financial literacy index that combines the knowledge, awareness, and numeracy indices. Was found an increase of 0.039 standard deviations in the education‐only treatment group (*p* = .078), an increase of 0.056 standard deviations in the education + account treatment group (*p* = .001), and no significant effect in the account‐only treatment.
IPA, Aflatoun, Ghana (Berry et al., 2015)	Financial education fund	5000 primary and junior high school students 6–14 years	Southern Ghana districts: 36 in Nkwanta, 30 in Greater Accra East, and 69 in Sekondi Takoradi Metropolitan Area. 135 primary and junior high schools	T1: Financial education and social education (personal exploration, child rights) plus locked money box with accompanied training T2: Only financial education plus locked money box with accompanied training C: No intervention	CRCT	9 months	+ Both treatments had a positive effect on savings behavior, mostly because students shifted their savings from home to school in a voluntary after‐school savings club with a locked money box − No changes in savings attitudes and financial literacy compared to control group + Few significant effects on risk preferences and spending − Financial education alone led children to work more, possibly shift from leisure or home production. However, school attendance did not change. No effect for integrated curriculum with money box. Also, differences are not statistically significant. − No treatment effects on child labor	HMB leading to an increase of 0.16 standard deviations (*SE* = 0.058), and Aflatoun producing a 0.12 standard deviation (*SE* = 0.053) increase. The difference between the Aflatoun and HMB program is not statistically significant (*p* = .48).
Suubi Project, Uganda (Ssewamala et al., 2009)	Grant from the National Institute of Mental Health	131 HIV‐orphaned adolescents' average age was 13.7 years at baseline (with a range from 11 to 17). Children were in their final year of primary school (which in Uganda goes up to grade 7), 57% girls	Rural Uganda Rakai District 15 primary schools	T: Family‐focused financial ed.; peer mentorship on life options; targeted child savings account plus counseling on life skills, health education and school supplies C: Typical orphan care (counseling on life skills, school supplies)	CRCT	10‐month follow‐up	+ Adolescents' self‐rated health, sexual risk‐taking behavior, and mental health functioning. Health and mental health functioning were found to be positively associated with each other.	2.15 (1.05, 4.42) The treatment group has over twice the odds of rating their health as good or excellent than the control group (OR = 2.15). Limitations: savings data only collected from treatment group.
Suubi Project, Uganda (Ssewamala & Ismayilova, 2009)	U.S. National Institute of Mental Health	135 HIV‐orphaned youths (ages 11–17), the average age was 13.7 years, 57% girls	Rakai district of Southern Uganda 15 rural primary schools	T: Family‐focused financial ed.; peer mentorship on life options; targeted child savings account plus counseling on life skills, health education and school supplies C: Typical orphan care (counseling on life skills, school supplies)	CRCT	10‐month follow‐up	+ Attitudes toward saving, + Attitudes toward academic performance + Attitudes toward educational aspirations + Sexual risk‐taking behavior and attitudes.	1) F(1, 257) = 27.91, *p* < .001, partial eta‐squared (δη^2^) = 0.1 2) F(1, 188) = 7.57, *p* < .01, partial eta‐squared (δη^2^) = 0.039 3) F(1, 225) = 4.52, *p* < .05, partial eta‐squared (δη^2^) = 0.025 4) F(1, 254) = 28.66, *p* < .001, partial eta‐squared (δη^2^) = 0.101
Suubi Project, Uganda (Ssewamala et al., 2010)	National Institute of Health	135 HIV‐orphaned adolescents The average age was 13.7 years, 60% girls in the treatment	Rakai district of Southern Uganda 15 rural primary schools	Same as above	RCT	10‐month follow‐up	+ Attitudes toward sexual risk for males = No change for females = Females and males saved comparable amounts	F(1,266) = 16.1, *p* < .0.001, partial eta‐squared (δη2) = 0.6, 95% CI
Suubi Project, Uganda (Ssewamala et al., 2012)	National Institute of Mental Health	138 HIV‐orphaned adolescents, specifically in primary six or primary seven (7th and 8th grade in the US education system), 57% of girls	Rakai district of Southern Uganda 15 primary schools	Same as above	RCT	10 and 20 month	+ Significant decrease in depression compared to control group (no change)	Depression among the HIV‐orphaned youth intervention group had a statistically significant reduction over time (B = −0.34, t(256) = −2.41, *p* = .02; 95% CI = (−0.61,−0.06)
Suubi Project, Uganda (Ssewamala et al., 2016)	National Institute of Mental Health	AIDS‐orphaned adolescents aged 12–16 years in the last 2 years of primary school. The average age was 13.4 years, 65% girls	Rakai and Masaka district of Uganda	Same as above	CRCT	24 month follow‐up	+ better educational outcomes, lower levels of hopelessness, and higher levels of self‐concept	Children in the Suubi‐Maka intervention group were 7.16 times more likely to take the Primary Leaving Examination (PLE) than children in the control group (OR = 7.16, 95% CI : 2.03, 25.29) children in the treatment group had much higher confidence in their educational plans than did their counterparts in the control group (OR = 6.06; 95% CI = 1.23, 29.91; *p* < .05). Also, children who, at baseline, reported having excellent physical health (OR = 4.35; 95% CI = 1.54, 12.26; *p* < .01) or good physical health (OR = 2.35; 95% CI = 1.16, 4.8; *p* < .05) had higher odds of being more confident in achieving their educational plans than children who reported having poor physical health.
Suubi‐Maki Project, Uganda (Karimli, 2013)	National Institute of Mental Health	179 AIDS‐orphaned adolescents (10–15 years old) and their parents	SouthwestUganda's Rakai and Masaka Districts 10 primary schools	T: Family‐focused financial ed.; peer mentorship on life options; targeted child savings account plus counseling on life skills, health education and school supplies C: Typical orphan care (counseling on life skills, school supplies)	CRCT	12 and 24 months	+ Adolescent's self‐reported savings significantly affected (effect weakened by number of children in the household) = Savings attitudes not affected However, savings attitudes found to be significantly affected by family relations, family finance, caregivers' gender, adolescent's gender, and educational aspirations.	Older adolescents were more likely to report having saved money (odds ratio = 1.35; 95% CI = 1.19, 1.53), and they reported saving more (B = 0.83; 95% CI = 0.34, 1.31) than their younger counterparts. Female adolescents were less likely to report having saved money (odds ratio = 0.48; 95% CI = 0.33, 0.7), and they reported saving less (B = −1.84; 95% CI = −3.01, −0.67) than their male counterparts. Similarly, adolescents with higher scores on the Beck Hopelessness Scale were less likely to report having saved money (odds ratio = 0.93; 95% CI = 0.88, 0.99), and they reported saving less (B = −0.18; 95% CI = −0.3, −0.06) than adolescents with lower scores on the Beck Hopelessness Scale. Also, participants who planned to start secondary school saved less (B = −1.03; 95% CI = −1.6, −0.46) than adolescents who did not plan to start secondary school.
Suubi‐Maka Project, Uganda (Karimli et al., 2014)	National Institute of Mental Health	179 HIV‐orphaned adolescents (10–15 years old) and their parents. The average age of study participants was 13 years, 65% of girls.	SouthwestUganda's Rakai and Masaka Districts 10 primary schools	T: Family‐focused financial ed.; peer mentorship on life options; targeted child savings account plus counseling on life skills, health education and school supplies C: Typical orphan care (counseling on life skills, school supplies)	CRCT	12 and 24 months	+ Increased likelihood of family reporting having money saved = No significant effect on the reported amounts saved = No significant effect of the use of formal financial institutions	Children in the treatment group were more likely to report having money saved both at wave 2 (odds ratio = 2.72; 95% Confidence Interval (CI) = 1.4, 5.29, *p* < .01) and at wave 3 (odds ratio = 2.26; 95% CI = 1.12, 4.57, *p* < .05).
Suubi‐Maka Project, Uganda (Jennings et al., 2016)	National Institute of Mental Health	346 orphaned adolescents, aged 10–17 years, on the last two years of primary school (equivalent to US 6th and 7th grades)	Rakai and Masaka districts	T: Family‐focused financial ed.; peer mentorship on life options; targeted child savings account plus counseling on life skills, health education and school supplies C: Typical orphan care (counseling on life skills, school supplies)	CRCT	12–24 moths	+ Significant relative increase over time in HIV‐preventive attitudinal scores in youth Had effect. + Significant increase on the savings behavior of vulnerable youth (cash savings over time)	Significant relative increase over time in HIV‐preventive attitudinal scores in youth (β = +0.19, ±0.09, *p* < .05), intervention had 2.017 significantly greater odds of a maximum HIV‐prevention score (OR = 2.017, 95% CI = 1.43,2.84), most commonly toward perceived risk of HIV (95.8%, n = 159), sexual abstinence or postponement (91.6%, n = 152) and consistent condom use (93.4%, n = 144). Had effect on the savings behavior of vulnerable youth; adolescents had an average significant increase of $US12.32 in CSA deposits per time period (β = 12.317, ±1.122, *p* < .001) and an average significant increase of $US15.00 in combined savings per time period (β = 14.996, ±1.657, *p* < .001), adjusting for changes over time in the control group.
Underage, Mongolia (Witte et al., 2015)	National Institute of Mental Health	107 Female street‐based sex workers at least 18 years. Average age 36 years. Most high school graduates, divorced, widowed, or separated.	Ulaanbaatar, Mongolia, In select districts	T: HIV sexual risk reduction training; matched savings; financial education (including negotiating); business training; mentoring C: HIV sexual risk reduction training Combined 4‐session	RCT	At 3‐ and 6‐month follow‐up assessments	+ 31% decrease number paying sexual partners + 39% decrease unprotected sexual contacts with paying partners	Participants exhibited a 31% decrease in the number of paying sexual partners at each time point, participants in the Undarga Program group exhibited a 22% greater decrease than those in HIVSRR‐alone group for each time point, participants in the Undarga Program group reported 50% fewer paying sexual partners at the 6‐month time point, participants exhibited a 39% decrease in the number of unprotected vaginal sex acts with paying partners at each time point.
SHAZ!, Zimbabwe (Dunbar et al., 2014)	National Institutes of Health	315 Adolescent female orphans, aged 16 to 19 were recruited through community out of school, not currently pregnant, HIV‐uninfected	Chitungwiza, a high density, urban area outside of Harare, Zimbabwe In Community	T: Mixed life skills, health education, financial education, vocational training (to start income‐generating activity). Following intervention, micro‐grants for business and social supports: Mixed life skills and health education	RCT	2‐year follow‐up every 6 months after for 24 months	+ Reduced food insecurity, improved economic situation + Lower risk of transactional sex + Higher likelihood of using condoms + Fewer unintended pregnancies	Reduced food insecurity (IOR = 0.83 vs. COR = 0.68, *p* = .02), and having their own income (IOR = 2.05 vs. COR = 1.67, *p* = 50.02), there was a lower risk of transactional sex (IOR = 0.64, 95% CI [0.50, 0.83]), and a higher likelihood of using a condom with their current partner (IOR = 1.79, 95% CI [1.23, 2.62]), there was also evidence of fewer unintended pregnancies among intervention participants (HR = 0.61, 95% CI [0.37, 1.01]), over time compared to baseline
Biruh Tesfa, Ethiopia (Erulkar et al., 2013)	The United States Agency for International Development (USAID) United Nations Population Fund (UNFPA), the Nike Foundation, the United Nations Foundation, and the Fisher Family Foundation also supported the project	640 Girls in slum out‐of‐school, and aged 12–19.	Addis Ababa, Bahir Dar, and Gondar, Ethiopia Kebele (sub‐district) community halls, mainly government‐run facilities	T: Mixed health education (including HIV awareness training, counseling, and testing), self‐ esteem, gender and power, basic literacy and financial education C: No intervention	Quasi‐Experimental (descriptive and multivariate)	30 months of implementation (2008–mid‐2011)	+ More than twice likely to report social support + Twice as likely to score highly on HIV knowledge questions + Know where to obtain voluntary counseling and testing + Want to be tested	Girls in the intervention sites were more than twice as likely to report social support (OR) = 2.0) compared to girls in the control site. They were also twice as likely (OR = 1.9) to score highly on HIV knowledge questions, to know where to obtain voluntary counseling and testing (OR = 2.0) and to want to be tested (OR = 1.9)
Kishoree Kontha Project, Bangladesh (Scales et al., 2013)	Nike Foundation	200 (2009) and 534 (2010) adolescent girls aged 10–19 years	Rural Bangladesh villages Babuganj, Muladi, Patuakhali Sadar, Bauphal, and Bhola Sadar), in three coastal districts (Barisal, Patuakhali, and Bhola) of the South Central region Spare household spaces that served as learning centers for the project girls	T1: Social skills, literacy, and school learning, health education. T2: Above plus financial education T3: Above plus nutrition incentive when marriage is delayed: No intervention	Experimental (Control group, villages randomly assigned to intervention or control)	6 and 9 month	+ Significant increase in external assets (support, empowerment, boundaries and expectations, constructive use of time) + Significant increase in internal assets (commitment to learning, positive values, social competencies, positive identity)	Combining the cohort data, these results suggest a mean improvement of 22% in the amount of developmental assets experienced, net of contamination and control group effects, and a mean net effect size of 0.80, conventionally considered a large effect
Reach, India (Spielberg et al., 2013)	Nike Foundation	2,227 Women members of women's self‐help groups (SHGs), adolescent daughters or daughters‐in‐law (10–19 years old) 547 (after 12 months)	Nadia West Bengal India 55 villages	T: Learning games with mixed health education (including sanitation, body, HIV protection) and financial education (including bargaining power) C: No intervention	RCT	6 and 12 month	+ Significant gains in HIV knowledge, awareness that condoms prevent HIV + Self‐efficacy for HIV prevention + Confirmed use of clean needles − Savings education showed no impact on financial knowledge or behavior change Limitation: Outcomes are not age or youth disaggregated	Girls had significantly higher motivation to save money (adjusted odds ratio 2.1, *p* = .002) and were more likely to have a plan to save money (adjusted odds ratio [OR] 2.2, *p* = .04). Girls who received HIV education had significant increases in HIV knowledge: heard of HIV (OR = 3.6, *p* < .05), heard of sexually transmitted infections (STIs) (OR = 3.6, *p* < .05), and aware that condoms can prevent HIV (OR = 2.9, *p* < .05).
BRAC, Uganda (Bandiera et al., 2012, 2015)	Bank Netherlands, Mastercard, Nike, the Gender Action Plan of the World Bank, Africa Gender Innovation Lab, the Improving Institutions for Pro‐Poor Growth program of DfID and the International Growth Centre	4,800 Girls 14–20 years. Average age is 16 years. 70% enrolled in school.	Kampala, Mukono, Iganga, and Jinja in Uganda Community clubs	T: Mixed health education (life skills to build knowledge and reduce risky behaviors), financial education (including negotiation skills and how to make use of financial services), vocational training (to start income‐generating activity) C: No intervention	RCT	2 and 4 years	+ Significantly improves HIV and pregnancy‐related knowledge + Significantly improves HIV and pregnancy‐related behaviors (sex against their will dropped 26%; early marriage and cohabitation fell by 58%; teen pregnancy fell by 26%) + Raised the likelihood of girls being engaged in income‐generating activities mainly driven by increased participation in self‐employment + Raised likelihood of engaging in income‐generation activity (2015) = No raised likelihood of engaging in income‐generating activity (2012)	ITT estimates: girls in treated communities are 6.8pp more likely to engage in income‐generating activities relative to girls in control communities, a 72% increase that is driven by additional engagement in self‐employment. 90% more likely to be self‐employed. Accompanied by monthly increases in consumption expenditures (38% from baseline) and significant reductions in job‐related anxieties. 7.2pp higher for treatment. Share of girls reporting sex against their will, early marriage and early childbearing drops

## APPENDIX 4 META‐ANALYSIS OF ODDS RATIOS GRAPH

**Odds ratios output table**

**Table:**

Study	OR	95% Confidence interval		% Weight
Ssewamala et al. (2009)	0.843	0.322	2.208	7.37
Ssewamala and Ismayilova (2009)	0.182	0.031	1.065	5.22
Ssewamala et al. (2010)	1.091	0.304	3.910	3.69
Ssewamala et al. (2012)	0.455	0.068	3.043	2.66
Karimli et al. (2014)	1.091	0.270	4.408	3.08
Jennings et al. (2016)	0.741	0.330	1.665	11.21
Ssewamala et al. (2016)	0.115	0.014	0.953	3.86
Dunbar et al. (2014)	0.434	0.174	1.083	11.29
Erulkar et al. (2013)	1.607	0.491	5.264	3.52
Berry et al. (2015)	1.894	1.224	2.931	22.85
Spielberg et al. (2013)	2.205	1.459	3.334	25.24
M‐H pooled OR	1.340	1.062	1.691	100.00

*Note*: Heterogeneity Chi‐squared = 28.38 (d.f. = 10) *p* = .002. I‐squared (variation in OR attributable to heterogeneity) = 64.8%. Test of OR = 1: z = 2.47 *p* = .014.

## References

[cl21071-bib-0001] Akwara, P. A. , Noubary, B. , Lim Ah Ken, P. , Johnson, K. , Yates, R. , Winfrey, W. , … Luo, C. (2010). Who is the vulnerable child? Using survey data to identify children at risk in the era of HIV and AIDS. AIDS Care, 22(9), 1066–1085. 20824560 10.1080/09540121.2010.498878

[cl21071-bib-0002] Bandiera, O. , Buehren, N. , Burgess, R. , Goldstein, M. , Gulesci, S. , Rasul, I. , & Sulaiman, M. (2012). Empowering adolescent girls: Evidence from a randomized control trial in Uganda. Washington, DC: World Bank.

[cl21071-bib-0003] Bandiera, O. , Buehren, N. , Burgess, R. , Goldstein, M. , Gulesci, S. , Rasul, I. , & Sulaimany, M. (2015, June). *Women's empowerment in action: Evidence from a randomized control trial in Africa* (Working Paper No. 50). Washington, DC: World Bank.

[cl21071-bib-0004] Bandura, A. (1977). Self‐efficacy: Toward a unifying theory of behavioral change. Psychological Review, 84(2), 191–215. 847061 10.1037//0033-295x.84.2.191

[cl21071-bib-0005] Bandura, A. (1994). Self‐efficacy. In V. S. Ramachaudran (Ed.), Encyclopedia of human behavior (4, pp. 71–81). New York: Academic Press. (Reprinted in H. Friedman [Ed.], (1998). *Encyclopedia of mental health*. San Diego: Academic Press). https://www.uky.edu/~eushe2/Bandura/Bandura1994EHB.pdf

[cl21071-bib-0006] Berry, J. , Karlan, D. , & Pradhan, M. (2015). *The impact of financial education for youth in Ghana* (Tingbergen Institute Discussion Paper 043‐V). Amsterdam, The Netherlands: Tinbergen Institute.

[cl21071-bib-0007] Cui, R. R. , Lee, R. , Thirumurthy, H. , Muessig, K. E. , & Tucker, J. D. (2013). Microenterprise development interventions for sexual risk reduction: A systematic review. AIDS and Behavior, 17(9), 2864–2877. 23963497 10.1007/s10461-013-0582-1 PMC3877769

[cl21071-bib-0008] Child and Youth Finance International (2012). *Children and youth as economic citizens: Review of research on financial capability, financial inclusion, and financial education* (Research Working Group Report). Amsterdam, The Netherlands: CYFI.

[cl21071-bib-0009] Department for International Development (DFID) (2014). Assessing the strength of evidence. London, UK: DFID.

[cl21071-bib-0010] De Guzman, A. (2001). Reducing social vulnerability to HIV/AIDS: models of care and their impact in resource‐poor settings. AIDS Care, 13(5), 663–675. 11571013 10.1080/09540120120063287

[cl21071-bib-0011] Dunbar, M. S. , Dufour, M. S. K. , Lambdin, B. , Mudekunye‐Mahaka, I. , Nhamo, D. , & Padian, N. S. (2014). The SHAZ! project: Results from a pilot randomized trial of a structural intervention to prevent HIV among adolescent women in Zimbabwe. PLoS One, 9(11):e113621. 25415455 10.1371/journal.pone.0113621 PMC4240618

[cl21071-bib-0012] Dworkin, S. L. , & Blankenship, K. (2009). Microfinance and HIV/AIDS prevention: Assessing its promise and limitations. AIDS and Behavior, 13(3), 462–469. 19294500 10.1007/s10461-009-9532-3 PMC3770268

[cl21071-bib-0013] Erulkar, A. , Ferede, A. , Girma, W. , & Ambelu, W. (2013). Evaluation of “Biruh Tesfa” (Bright Future) program for vulnerable girls in Ethiopia. Vulnerable Children and Youth Studies, 8(2), 182–192.

[cl21071-bib-0014] Fleischman, J. , & Peck, K. (2015). Addressing HIV risk in adolescent girls and young women. Washington, DC: Center for Strategic and International Studies.

[cl21071-bib-0015] International Center for Research on Women (2010). What role can economic empowerment strategies play in reducing HIV risk and vulnerability among girls and young women? Washington, DC: International Center for Research on Women.

[cl21071-bib-0016] Jamison, J. C. , Karlan, D. , & Zinman, J. (2014). *Financial education and access to savings accounts: Complements or substitutes? Evidence from Ugandan youth clubs* (National Bureau of Economic Research Working Paper No. 20135, JEL No. D12, D91, O12). Cambridge, MA: National Bureau of Economic Research.

[cl21071-bib-0017] Jennings, L. , Ssewamala, F. M. , & Nabunya, P. (2016). Effect of savings‐led economic empowerment on HIV preventive practices among orphaned adolescents in rural Uganda: Results from the Suubi‐Maka randomized experiment. AIDS Care, 28(3), 273–282. 26548549 10.1080/09540121.2015.1109585 PMC4747687

[cl21071-bib-0018] Kalamar, A. M. , Bayer, A. M. , & Hindin, M. J. (2016). Interventions to prevent sexually transmitted infections, including HIV, among young people in low‐ and middle‐income countries: A systematic review of the published and gray literature. Journal of Adolescent Health, 59(3), S22–S31. 10.1016/j.jadohealth.2016.05.020 27562450

[cl21071-bib-0019] Karimli, L. (2013). Financial asset accumulation by poor adolescents participating in child savings accounts in low resource communities in Uganda. New York, NY: Columbia University.

[cl21071-bib-0020] Karimli, L. , Ssewamala, F. M. , & Neilands, T. B. (2014). Poor families striving to save in matched children's savings accounts: Findings from a randomized experimental design in Uganda. Social Service Review, 88(4), 658–694. 25525282 10.1086/679256 PMC4267259

[cl21071-bib-0021] Kennedy, C. E. , Fonner, V. A. , O'Reilly, K. R. , & Sweat, M. D. (2014). A systematic review of income generation interventions, including microfinance and vocational skills training, for HIV prevention. AIDS Care, 26(6), 659–673. 24107189 10.1080/09540121.2013.845287 PMC3943565

[cl21071-bib-0022] Kim, J. , Pronyk, P. , Barnett, T. , & Watts, C. (2008). Exploring the role of economic empowerment in HIV prevention. AIDS, 22(Suppl 4), S57–S71. 10.1097/01.aids.0000341777.78876.40 19033756

[cl21071-bib-0023] Orton, L. , Pennington, A. , Nayak, S. , Sowden, A. , White, M. , & Whitehead, M. (2016). Group‐based microfinance for collective empowerment: A systematic review of health impacts. Bulletin of the World Health Organization, 94(9), 694–704. 27708475 10.2471/BLT.15.168252 PMC5034638

[cl21071-bib-0024] President's Emergency Plan for AIDS Relief (PEPFAR) (2012). Guidance for orphans and vulnerable children programming. Washington, DC: U.S. President's Emergency Plan for AIDS Relief. Retrieved from http://www.pepfar.gov/documents/organization/195702.pdf

[cl21071-bib-0025] Plourde, K. F. , Fischer, S. , Cunningham, J. , Brady, K. , & McCarraher, D. R. (2016). Improving the paradigm of approaches to adolescent sexual and reproductive health. Reproductive Health, 13(1), 72. 27296400 10.1186/s12978-016-0191-3 PMC4907011

[cl21071-bib-0026] Pronyk, P. M. , Kim, J. C. , Abramsky, T. , Phetla, G. , Hargreaves, J. R. , Morison, L. A. , … Porter, J. D. (2008). A combined microfinance and training intervention can reduce HIV risk behaviour in young female participants. AIDS, 22(13), 1659–1665. 18670227 10.1097/QAD.0b013e328307a040

[cl21071-bib-0027] Scales, P. C. , Benson, P. L. , Dershem, L. , Fraher, K. , Makonnen, R. , Nazneen, S. , … Titus, S. (2013). Building developmental assets to empower adolescent girls in rural Bangladesh: Evaluation of project kishoree kontha. Journal of Research on Adolescence, 23(1), 171–184.

[cl21071-bib-0028] Shephard, D. D. , Contreras, J. M. , Meuris, J. , te Kaat, A. , Bailey, S. , Custers, A. , & Spencer, N. (2017). Beyond financial Literacy: The psychological dimensions of financial capability. Think Forward Initiative. Retrieved from https://8b109582833d5c2e19d5‐b8e01d380645fda9dfa9d12a21c5e59a.ssl.cf3.rackcdn.com/downloads/Beyond‐financial‐literacy_The‐psychological‐dimensions‐of‐financial‐capability_Technical‐report.pdf

[cl21071-bib-0029] Spielberg, F. , Crookston, B. T. , Chanani, S. , Kim, J. , Kline, S. , & Gray, B. L. (2013). Leveraging microfinance to impact HIV and financial behaviors among adolescents and their mothers in West Bengal: A cluster randomized trial. International Journal of Adolescent Medicine and Health, 25(2), 157–166. 23324373 10.1515/ijamh-2013-0024

[cl21071-bib-0030] Ssewamala, F. M. , Han, C. K. , & Neilands, T. B. (2009). Asset ownership and health and mental health functioning among AIDS‐orphaned adolescents: Findings from a randomized clinical trial in rural Uganda. Social Science & Medicine, 69(2), 191–198. 19520472 10.1016/j.socscimed.2009.05.019 PMC2819297

[cl21071-bib-0031] Ssewamala, F. M. , & Ismayilova, L. (2009). Integrating children's savings accounts in the care and support of orphaned adolescents in rural Uganda. Social Service Review, 83(3), 453–472. 20445763 10.1086/605941 PMC2863345

[cl21071-bib-0032] Ssewamala, F. M. , Ismayilova, L. , McKay, M. , Sperber, E. , Bannon, W., Jr. , & Alicea, S. (2010). Gender and the effects of an economic empowerment program on attitudes toward sexual risk‐taking among AIDS‐orphaned adolescent youth in Uganda. Journal of Adolescent Health, 46(4), 372–378. 10.1016/j.jadohealth.2009.08.010 PMC2844862 20307827

[cl21071-bib-0033] Ssewamala, F. , Karimili, L. , Torsten, N. , Wang, J. S.‐H. , Han, C.‐K. , Ilic, V. , & Nabunya, P. (2016). Applying a family‐level economic strengthening intervention to improve education and health related outcomes of school‐going AIDS‐orphaned children: Lessons from a randomized experiment in Southern Uganda. Society for Prevention Research, 17(2016), 134–143. http://pubmedcentralcanada.ca/pmcc/articles/PMC4697878/ 10.1007/s11121-015-0580-9 PMC4697878 26228480

[cl21071-bib-0034] Ssewamala, F. M. , Neilands, T. B. , Waldfogel, J. , & Ismayilova, L. (2012). The impact of a comprehensive microfinance intervention on depression levels of AIDS‐orphaned children in Uganda. Journal of Adolescent Health, 50(4), 346–352. 10.1016/j.jadohealth.2011.08.008 PMC3314188 22443837

[cl21071-bib-0035] UNAIDS (2015). Update: Active involvement of young people is key to ending the AIDS epidemic by 2030. Geneva, Switzerland: UNAIDS.

[cl21071-bib-0036] UNAIDS (2016). Global Aids Update 2016. Geneva, Switzerland: UNAIDS.

[cl21071-bib-0037] UNAIDS & The African Union (2015). Empowerment young women and adolescent girls: Fast‐tracking the end of the aids epidemic in Africa. Geneva, Switzerland: UNAIDS and the African Union.

[cl21071-bib-0038] UNICEF (2016). State of the world's children. New York, NY: UNICEF.

[cl21071-bib-0039] van der Knaap, L. M. , Leeuw, F. L. , Bogaerts, S. , & Nijssen, L. T. J. (2008). Combining campbell standards and the realist evaluation approach: The best of two worlds? American Journal of Evaluation, 29(1), 48–57.

[cl21071-bib-0040] World Health Organization (WHO) (2014). Health for the world's adolescents: A second chance in the second decade. Geneva: World Health Organization.

[cl21071-bib-0041] Witte, S. S. , Aira, T. , Tsai, L. C. , Riedel, M. , Offringa, R. , Chang, M. , … Ssewamala, F. (2015). Efficacy of a savings‐led microfinance intervention to reduce sexual risk for HIV among women engaged in sex work: A randomized clinical trial. American Journal of Public Health, 105(3), e95–e102. 10.2105/AJPH.2014.302291 PMC4330842 25602889

[cl21071-bib-0042] YouthPower (2017). A systematic review of positive youth development programs in low‐and middle‐income countries. Washington, DC: YOUTHPOWER.

[cl21071-bib-0043] Amin, S. , Rahman, L. , Ainul, S. , Rob, U. , Zaman, B. , & Akter, R. (2010). Enhancing adolescent financial capabilities through financial education in Bangladesh. Dhaka: Population Council

[cl21071-bib-0044] Baxter, A. , Chapman, D. W. , DeJaeghere, J. , Pekol, A. R. , & Weiss, T. (2014). Youth entrepreneurship education and training for poverty alleviation: A review of international literature and local experiences. International Educational Innovation and Public Sector Entrepreneurship, 23, 33–58.

[cl21071-bib-0045] Birkenmaier, J. , & Maynard, B. (2016). Interventions designed to improve financial capability by improving financial behaviour and financial access: A systematic review. The Campbel Collaboration, 1–12. 10.1002/cl2.1020 PMC8356530 37131461

[cl21071-bib-0046] Bruce, J. , & Hallman, K. (2008). Reaching the girls left behind. Gender & Development, 16(2), 227–245.

[cl21071-bib-0047] Fernandes, D. , Lynch, J. G., Jr. , & Netemeyer, R. G. (2014). Financial literacy, financial education, and downstream financial behaviors. Management Science, 60(8), 1861–1883.

[cl21071-bib-0048] Gibbs, A. , Willan, S. , Misselhorn, A. , & Mangoma, J. (2012). Combined structural interventions for gender equality and livelihood security: A critical review of the evidence from southern and eastern Africa and the implications for young people. Journal of the International AIDS Society, 15(Suppl 1), 17362. 10.7448/IAS.15.3.17362 PMC3499786 22713350

[cl21071-bib-0049] Hastings, J. S. , Madrian, B. C. , & Skimmyhorn, W. L. (2013). Financial literacy, financial education, and economic outcomes. Annual Review of Economics, 5, 347–373. 10.1146/annurev-economics-082312-125807 PMC3753821 23991248

[cl21071-bib-0050] Kaiser, T. , & Menkhoff, L. (2016). *Does financial education impact financial behavior, and if so, when?* (Discussion Paper No. 1562). Berlin: Deutsches Institut für Wirtschaftsforschung.

[cl21071-bib-0051] Kalamar, A. M. , Lee‐Rife, S. , & Hindin, M. J. (2016). Interventions to prevent child marriage among young people in low‐ and middle‐income countries: A systematic review of the published and gray literature. Journal of Adolescent Health, 59, S16–S21. 10.1016/j.jadohealth.2016.06.015 27562449

[cl21071-bib-0052] Karlan, D. , Ratan, A. L. , & Zinman, J. (2014). Savings by and for the poor: A research review and agenda. Review of Income and Wealth, 60(1), 36–78. 25792764 10.1111/roiw.12101 PMC4358152

[cl21071-bib-0053] Lundberg, M. & Mulaj, F. (Eds.), 2014). Enhancing Financial Capability And Behavior In Low‐ And Middle‐Income Countries. Washington: World Bank.

[cl21071-bib-0054] Massey, E. , Wyatt, A. , & Smit, C. (2016). Evaluating financial education initiatives in South Africa: The importance of multiple evaluation approaches. African Evaluation Journal, 4(1), 1–6.

[cl21071-bib-0055] McCormick, M. H. (2009). The effectiveness of youth financial education: A review of the literature. Journal of Financial Counseling and Planning, 20(1), 70–83.

[cl21071-bib-0056] Miller, M. , Reichelstein, J. , Salas, C. , & Zia, B. (2015). *Can you help someone become financially capable? A meta‐analysis of the literature* (Policy Research Paper No. 6745). Washington DC: World Bank.

[cl21071-bib-0057] O'Prey, L. , & Shephard, D. (2014). *Financial education for children and youth: A systematic review and meta‐analysis* (Working Paper No. 2014A).

[cl21071-bib-0058] OECD/INFE (2013). *Financial Education in Africa and Latin America*. Africa and Latin America: OECD/INFE Peer Review and Regional Reports.

[cl21071-bib-0059] Refera, M. K. , Dhaliwal, N. K. , & Kaur, J. (2016). Financial literacy for developing countries in Africa: A review of concept, significance and research opportunities. Journal of African Studies and Development, 8(1), 1–12.

[cl21071-bib-0060] Singh, J. , & Schneiders, M. (2017). A global desk review of financial education's contribution to girls' economic empowerment. Netherland: Aflatoun International.

[cl21071-bib-0061] Ssewamala, F. M. (2015). Optimizing the “demographic dividend” in young developing countries: The role of contractual savings and insurance for financing education. International Journal of Social Welfare, 24, 248–262. 26273211 10.1111/ijsw.12131 PMC4528970

[cl21071-bib-0062] Xu, L. , & Zia, B. (2013). *Financial literacy around the world. an overview of the evidence with practical suggestions for the way forward* (Policy Research Working Paper No. 6107). Washington DC: World Bank.

